# A Comparative Analysis of the Methylation Status of Non-Coding RNA Promoters in Fibroid and Matched Myometrium

**DOI:** 10.3390/ijms27177781

**Published:** 2026-08-31

**Authors:** Tsai-Der Chuang, Shawn Rysling, Abigail Wiseman, Gabriela Alfaro, Sayna Pejouhesh Jahromi, Sepideh Pejouhesh Jahromi, Daniel Baghdasarian, Omid Khorram

**Affiliations:** 1 The Lundquist Institute for Biomedical Innovation, Torrance, CA 90502, USA; tchuang@lundquist.org (T.-D.C.); srysling@gmail.com (S.R.); abigail.e.wiseman@gmail.com (A.W.); alfarogabrielamed@gmail.com (G.A.); s.pejouheshjahromi@lundquist.org (S.P.J.); s.pejouhesh-jahromi@lundquist.org (S.P.J.); 2 Department of Obstetrics and Gynecology, Harbor-UCLA Medical Center, Torrance, CA 90502, USA; dbaghdasarian@dhs.lacounty.gov; 3 Department of Obstetrics and Gynecology, David Geffen School of Medicine at University of California, Los Angeles (UCLA), Los Angeles, CA 90095, USA

**Keywords:** LncRNA, SncRNA, leiomyoma, fibroid, epigenetic modification, promoter methylation

## Abstract

Uterine fibroids exhibit dysregulated expression of non-coding RNAs (ncRNAs), although the underlying mechanisms remain incompletely understood. We investigated promoter DNA methylation and its relationship with ncRNA expression in fibroids. Genomic DNA from eight paired fibroid and matched myometrial tissues was analyzed using MeDIP–chip to identify differentially methylated ncRNA promoters. Selected candidates were validated by methylation-specific PCR (MSP) in 16 paired samples, and transcript expression was assessed by qRT-PCR in 68–94 paired specimens. MeDIP–chip identified 538 lncRNAs and 61 miRNAs with differential promoter methylation, including 300 hypermethylated and 238 hypomethylated lncRNAs and 47 hypermethylated and 14 hypomethylated miRNAs. Promoter methylation was not significantly correlated with transcript expression (r = −0.1224). MSP confirmed hypermethylation of LINC-PINT and MIR9-3 and hypomethylation of WT1-AS and TTLL10-AS1. Correspondingly, LINC-PINT and MIR9-3 expression was decreased, whereas WT1-AS and TTLL10-AS1 expression was increased in fibroids. However, LINC-PINT and TTLL10-AS1 methylation did not fully correspond with MeDIP–chip findings. These results reveal widespread ncRNA promoter methylation alterations in uterine fibroids but demonstrate that genome-wide methylation does not consistently predict transcript expression, highlighting the complexity of ncRNA epigenetic regulation and the importance of locus-specific validation.

## 1. Introduction

Uterine fibroids impact nearly 70% of women during their reproductive years, yet the molecular mechanisms driving fibroid initiation and development, which are characterized by pain, infertility, and abnormal bleeding, resulting in substantial morbidity and healthcare burden, remain largely unknown [1,2,3]. Uterine fibroids exhibit extensive epigenetic dysregulation, which contributes to altered gene expression without changes in DNA sequence. Beyond protein-coding genes, accumulating evidence indicates that non-coding RNAs (ncRNAs), including long non-coding RNAs (lncRNAs) and microRNAs (miRNAs), are epigenetically regulated and play critical roles in fibroid pathogenesis [4]. We and others have identified multiple miRNAs and lncRNAs that exert significant control over fibroid-associated gene networks [5,6,7,8,9,10]; these ncRNAs influence key cellular processes such as extracellular matrix (ECM) production, cell proliferation, inflammation, and hormone responsiveness, which are hallmark features of fibroids.

The inverse relationship between promoter DNA methylation and transcriptional activity is a fundamental principle of epigenetic gene regulation and has been extensively documented across mammalian systems [11,12]. Mammalian gene promoters display substantial heterogeneity in CpG density and GC content, which strongly influences their susceptibility to DNA methylation and their regulatory mechanisms. Genome-wide studies have demonstrated widespread DNA methylation alterations in fibroids compared with matched myometrium, including both global hypomethylation and locus-specific hypermethylation at gene promoters and regulatory regions [13,14]. Importantly, aberrant methylation patterns are not limited to protein-coding genes but also extend to ncRNA promoters, suggesting that epigenetic mechanisms contribute directly to ncRNA dysregulation in fibroids.

Based on CpG observed-to-expected ratios, GC content, and CpG-rich region length, promoters are commonly classified into high-CpG-density promoters (HCPs), low-CpG-density promoters (LCPs), and intermediate-CpG-density promoters (ICPs) [15,16]. HCPs, typically associated with CpG islands, contain a ≥500 bp region near the transcription start site with high GC content and CpG density. In embryonic stem cells, over 95% of HCPs remain unmethylated and are regulated primarily through chromatin-based mechanisms involving Trithorax-group (TrxG) and Polycomb-group (PcG) proteins, marked by H3K4me3 and H3K27me3, respectively [17,18]. These complexes are thought to be recruited, at least in part, via non-specific binding to unmethylated CpG-rich DNA. During differentiation, selective hypermethylation of CpG-dense promoters results in exclusion of TrxG/PcG activity, chromatin compaction, and stable transcriptional silencing [19]. Beyond DNA methylation, histone modifications and chromatin remodeling complexes also influence ncRNA regulation in fibroids. Alterations in histone methylation marks such as H3K27me3 and H3K4me3, mediated by Polycomb and Trithorax group proteins, have been implicated in fibroid-specific gene expression patterns [20]. These chromatin states may interact with DNA methylation to fine-tune ncRNA transcription. In contrast, LCPs lack CpG-rich regions and exhibit little correlation between promoter methylation status and transcriptional activity. Most LCPs are methylated regardless of expression state, indicating that low CpG density reduces the functional impact of cytosine methylation [15]. These promoters are often transcriptionally inactive by default and are selectively activated by cell type-specific transcription factors, rather than through PcG-mediated repression. ICPs represent a distinct and particularly methylation-sensitive promoter class. These promoters are preferential targets of de novo DNA methylation in somatic cells, and methylation effectively prevents their transcriptional activation [21,22]. ICP methylation has been proposed as a mechanism to restrict alternative lineage programs by selectively silencing differentiation-associated genes [23]. Their heightened susceptibility to methylation may reflect intermediate CpG density that fails to efficiently recruit protective chromatin modifiers, rendering ICPs especially vulnerable to epigenetic dysregulation in cancer and other disease states [24,25].

Several fibroid-associated lncRNAs are regulated by promoter methylation. H19, one of the most extensively studied lncRNAs in fibroids, is epigenetically controlled through imprinting and DNA methylation and is consistently overexpressed in fibroids, where it promotes cell proliferation and ECM accumulation [26,27]. Similarly, XIST, a key regulator of X-chromosome inactivation, shows aberrant expression in fibroids, potentially reflecting altered epigenetic states associated with X-linked gene dosage and chromatin remodeling [28]. MIAT has also been reported to be dysregulated in fibroids and other smooth muscle tumors, where it modulates cell growth and fibrotic signaling pathways [29]. MiRNAs in fibroids are likewise subject to epigenetic control. Promoter hypermethylation has been shown to suppress tumor-suppressive miRNAs, including members of the miR-29, miR-200, and let-7 families, which normally inhibit collagen synthesis, TGF-β signaling, and fibrotic gene expression [4]. Epigenetic silencing of these miRNAs contributes to excessive ECM deposition and fibroid growth, while demethylating agents can partially restore their expression in vitro.

Recent studies have established somatic MED12 mutations as one of the major molecular drivers of uterine fibroid pathogenesis [30,31]. MED12 is an essential component of the Mediator complex, a multiprotein transcriptional coactivator composed of approximately 30 subunits that regulates RNA polymerase II-dependent gene transcription [32,33]. Dysregulation of MED12 has been implicated in the development of numerous human malignancies [33]. In uterine fibroids, somatic mutations affecting exon 2 of MED12 occur in up to 80% of tumors and are thought to contribute to tumor initiation and progression through disruption of multiple signaling pathways, including Hedgehog, transforming growth factor-β (TGF-β), sex steroid receptor, and Wnt/β-catenin signaling [31,34,35,36,37]. Consistent with these observations, our previous transcriptomic studies demonstrated that the expression of both protein-coding and non-coding RNAs in uterine fibroids is influenced by patient race/ethnicity and MED12 mutation status, highlighting the complex interaction between genetic and epigenetic factors in fibroid biology [9,38,39].

The present study aimed to characterize genome-wide promoter methylation patterns of ncRNAs in uterine fibroids and to evaluate their relationship with ncRNA expression. Using MeDIP–chip microarray analysis together with methylation-specific PCR and expression validation, we sought to determine whether promoter methylation is associated with altered ncRNA expression and to identify candidate ncRNAs that may be subject to epigenetic regulation. Addressing this gap is essential for understanding fibroid biology and for identifying novel biomarkers and therapeutic targets, particularly for non-hormonal and disease-modifying treatments.

## 2. Results

### 2.1. MeDIP–Chip Analysis Reveals Differential Promoter Methylation of LncRNA and miRNA Between Fibroid and Paired Myometrium

**Figure 1:**
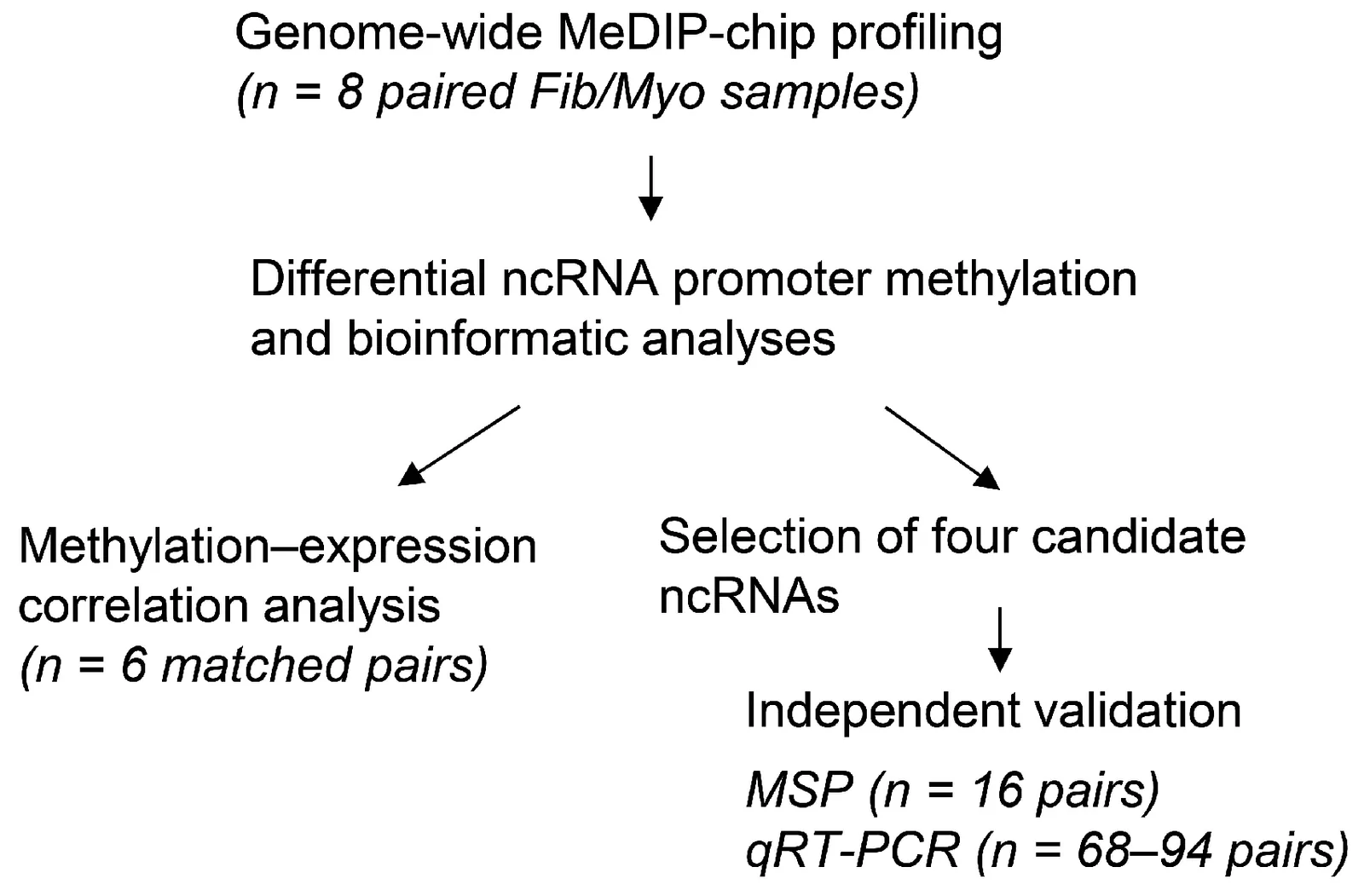
Overview of the study design and validation strategy. Genome-wide promoter methylation profiling was performed using MeDIP–chip in eight paired fibroid (Fib) and matched myometrial (Myo) samples, followed by identification and bioinformatic analysis of differentially methylated ncRNA promoters. Methylation–expression integration was performed using MeDIP–chip and gene-level RNA-seq data available from the same six paired Fib/Myo specimens. Four candidate ncRNAs were selected for independent validation of promoter methylation by methylation-specific PCR (MSP; *n* = 16 pairs) and transcript expression by qRT-PCR (*n* = 68–94 pairs).

**Figure 2:**
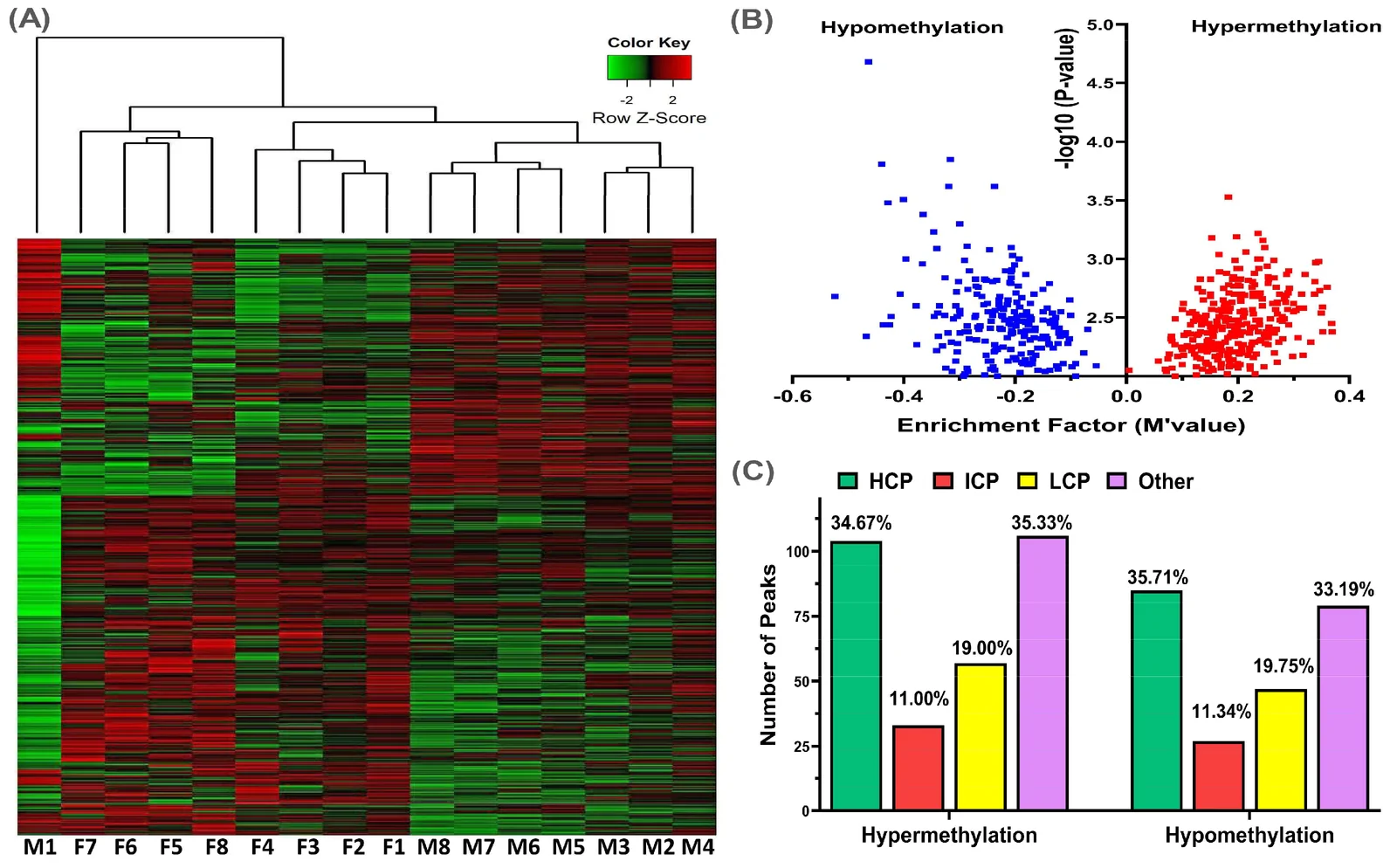
Differential promoter methylation of ncRNAs in fibroids and matched myometrium. (**A**) Hierarchical clustering heatmap showing differentially enriched promoter methylation peaks of ncRNAs in fibroids (F) compared with paired myometrium (M) (−log_10_ *p* ≥ 2; *n* = 8 pairs). The color scale represents relative enrichment levels expressed as z-scores. (**B**) Volcano plot illustrating differentially enriched promoter methylation peaks of ncRNAs in fibroids relative to matched myometrium. (**C**) Distribution of differentially enriched methylation peaks across promoter CpG-density categories identified in the microarray analysis. Promoters were classified as high CpG-density promoters (HCP), intermediate CpG-density promoters (ICP), or low CpG-density promoters (LCP).

An overview of the study design and validation strategy is shown in Figure 1. Genome-wide ncRNA promoter methylation was initially profiled by MeDIP–chip in eight paired fibroid and matched myometrial samples, followed by bioinformatic analyses of differentially methylated ncRNA promoters. Methylation and gene-level RNA-seq expression data from six matched pairs were integrated to assess the relationship between promoter methylation and transcript expression. Four candidate ncRNAs were subsequently selected for independent validation of promoter methylation by MSP (*n* = 16 pairs) and expression by qRT-PCR (*n* = 68–94 pairs). Firstly, genomic DNA was extracted from paired fibroid and matched myometrial tissues (*n* = 8 pairs) and analyzed for 5-methylcytosine (5mC) enrichment in lncRNA and miRNA promoter regions using the MeDIP–chip microarray. Following data normalization, hierarchical clustering and TreeView analyses identified 538 differentially methylated lncRNA promoter entries and 61 miRNA promoter regions in fibroids compared with matched myometrium (−log_10_ *p* ≥ 2) (Figure 2A,B and Supplementary Table S1). Among these, 238 lncRNA promoter entries (44.2%) and 14 miRNA promoter regions (23.0%) were hypomethylated, whereas 300 lncRNA promoter entries (55.8%) and 47 miRNA promoter regions (77.0%) were hypermethylated in fibroids relative to myometrium. Promoter classification by CpG density showed that among hypermethylated transcripts, 34.7% were HCPs, 11.0% ICPs, and 19.0% LCPs, while hypomethylated transcripts comprised 35.7% HCPs, 11.3% ICPs, and 19.8% LCPs (Figure 2C). These findings demonstrate widespread alterations in ncRNA promoter methylation in uterine fibroids.

### 2.2. Pathway Analysis of lncRNA and miRNA with Differential Promoter Methylation in Fibroid Reveals Affected Pathways

**Figure 3:**
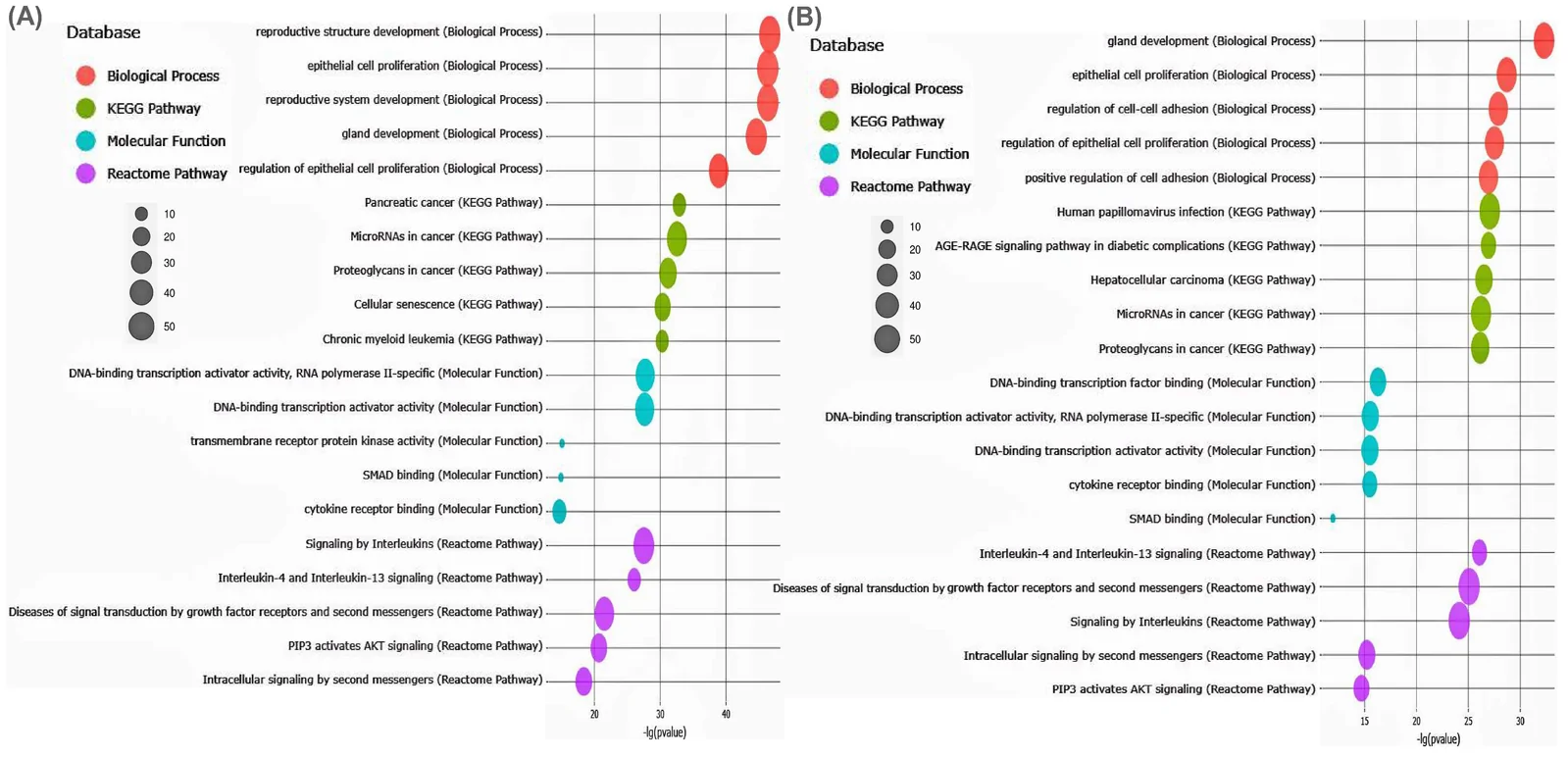
Functional enrichment analysis of differentially methylated lncRNAs in fibroids. Functional enrichment analyses, including Gene Ontology Biological Process (BP), KEGG pathway, Molecular Function (MF), and Reactome pathway analyses, were performed for hypomethylated (*n* = 238) and hypermethylated (*n* = 300) lncRNAs identified from promoter methylation analysis in fibroids compared with matched myometrium. The enriched pathways for hypomethylated lncRNAs (**A**) and hypermethylated lncRNAs (**B**) are shown. The circle size represents the number of genes associated with each enriched pathway.

**Figure 4:**
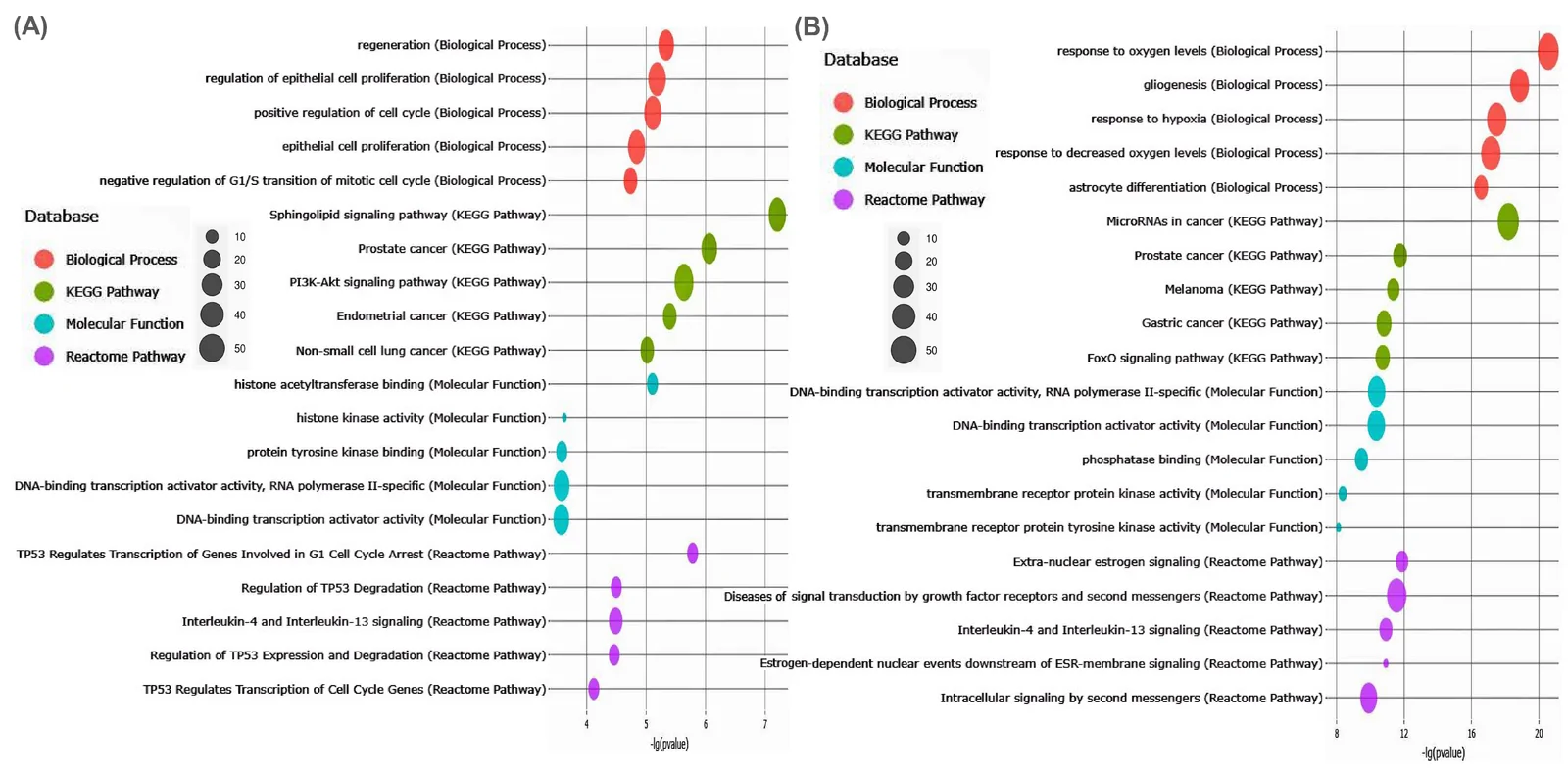
Functional enrichment analysis of differentially methylated miRNAs in fibroids. Functional enrichment analyses, including Gene Ontology Biological Process (BP), KEGG pathway, Molecular Function (MF), and Reactome pathway analyses, were performed for hypomethylated (*n* = 14) and hypermethylated (*n* = 47) miRNAs identified from promoter methylation analysis in fibroids compared with matched myometrium. The enriched pathways for hypomethylated miRNAs (**A**) and hypermethylated miRNAs (**B**) are shown. The circle size represents the number of genes associated with each enriched pathway.

Functional enrichment analyses (Biological Process, KEGG, Molecular Function, and Reactome) were performed on the 238 hypomethylated and 300 hypermethylated lncRNAs identified in fibroids (Figure 3A,B). Hypomethylated lncRNAs were primarily enriched in pathways related to miRNAs in cancer, proteoglycans in cancer, and cellular senescence, whereas hypermethylated lncRNAs were most strongly associated with HPV infection, miRNAs in cancer, and proteoglycan-related pathways. Similarly, enrichment analysis of the 14 hypomethylated and 47 hypermethylated miRNAs (Figure 4A,B) revealed that hypomethylated miRNAs were predominantly linked to the sphingolipid signaling pathway, PI3K/AKT signaling, and histone acetyltransferase binding, while hypermethylated miRNAs were enriched in miRNAs in cancer, FoxO signaling, and extranuclear estrogen signaling pathways. Collectively, these data suggest that ncRNAs with altered promoter methylation are associated with cancer-related, hormone-responsive, and PI3K/AKT-associated signaling networks relevant to fibroid pathogenesis.

### 2.3. qPCR and Methylation-Specific PCR Validate Array Data

**Figure 5:**
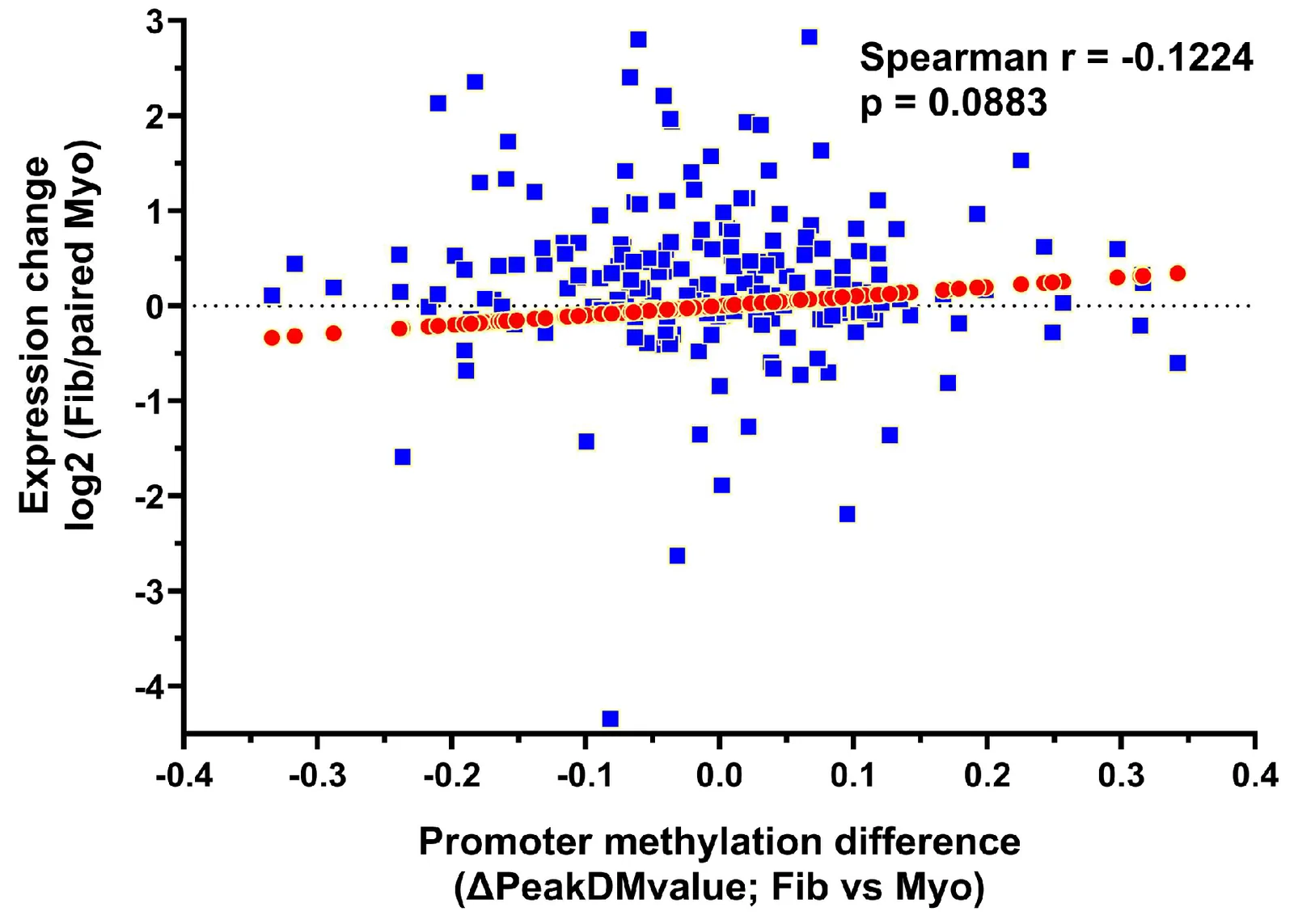
Correlation between promoter methylation difference and ncRNA expression change in fibroids. The *x*-axis shows promoter methylation differences (ΔPeakDMvalue; Fib vs. Myo; red) obtained from MeDIP–chip analysis, while the *y*-axis shows ncRNA expression changes expressed as log_2_ fold change (Fib/paired Myo; blue) derived from RNA-seq data derived from the same paired specimens (*n* = 6 pairs). Each point represents an individual ncRNA. Spearman correlation analysis indicated no significant association between promoter methylation and transcript expression (r = −0.1224, *p* = 0.0883), suggesting that promoter methylation alone does not uniformly predict ncRNA expression in fibroids.

We next examined the relationship between promoter methylation and transcript expression by integrating the MeDIP–chip data (GSE324138) with our previously published RNA-seq datasets (GSE224991 and GSE289632) derived from the same paired specimens (*n* = 6 pairs). Because MeDIP–chip promoter annotations are transcript-specific, whereas the available RNA-seq dataset was quantified at the gene level, the present correlation analysis reflects the relationship between promoter methylation and corresponding gene-level expression rather than transcript isoform-specific expression. Correlation analysis revealed no significant association between promoter methylation status and ncRNA expression levels in fibroids compared with matched myometrium (Spearman r = −0.1224, *p* = 0.0883; Figure 5 and Supplementary Table S2).

For directional methylation–expression analysis, ncRNAs meeting the predefined RNA-seq differential-expression threshold (fold change ≥ 1.5 or ≤0.67 in fibroid versus matched myometrium) were classified according to the direction of promoter methylation and expression. An inverse relationship was defined as promoter hypermethylation accompanied by decreased expression or promoter hypomethylation accompanied by increased expression. Loci not meeting the RNA-seq differential-expression threshold were not included in the directional classification. Among the 69 loci meeting the predefined RNA-seq differential-expression criterion (fold change ≥ 1.5 or ≤0.67), 40 (58.0%) exhibited an inverse methylation–expression relationship, including 29 showing promoter hypomethylation with increased expression and 11 showing promoter hypermethylation with decreased expression. The remaining 29 loci (42.0%) exhibited non-inverse relationships (Supplementary Table S2).

**Figure 6:**
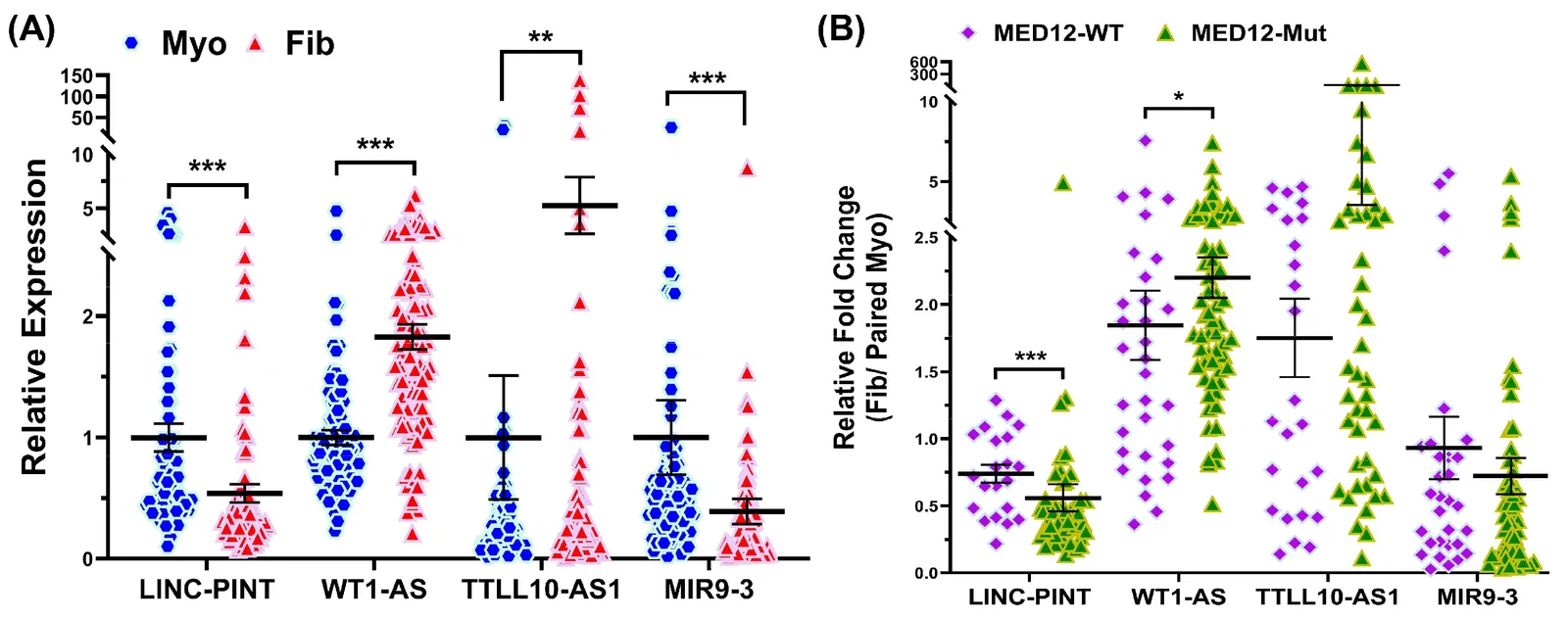
Validation of ncRNA expression in fibroids and association with MED12 mutation status. (**A**) Relative expression levels of LINC-PINT (*n* = 68 pairs), WT1-AS (*n* = 94 pairs), TTLL10-AS1 (*n* = 70 pairs), and MIR9-3 (*n* = 86 pairs) measured by qRT-PCR in paired myometrium and fibroid tissues. (**B**) Expression levels of the same ncRNAs, expressed as fold change (fibroid/paired myometrium), were further analyzed according to MED12 mutation status, including MED12 mutated (*n* = 47–62) and MED12 wild-type (*n* = 21–32) specimens. Data are presented as mean ± SEM, and statistical significance is indicated by connecting lines (* *p* < 0.05; ** *p* < 0.01; *** *p* < 0.001).

**Figure 7:**
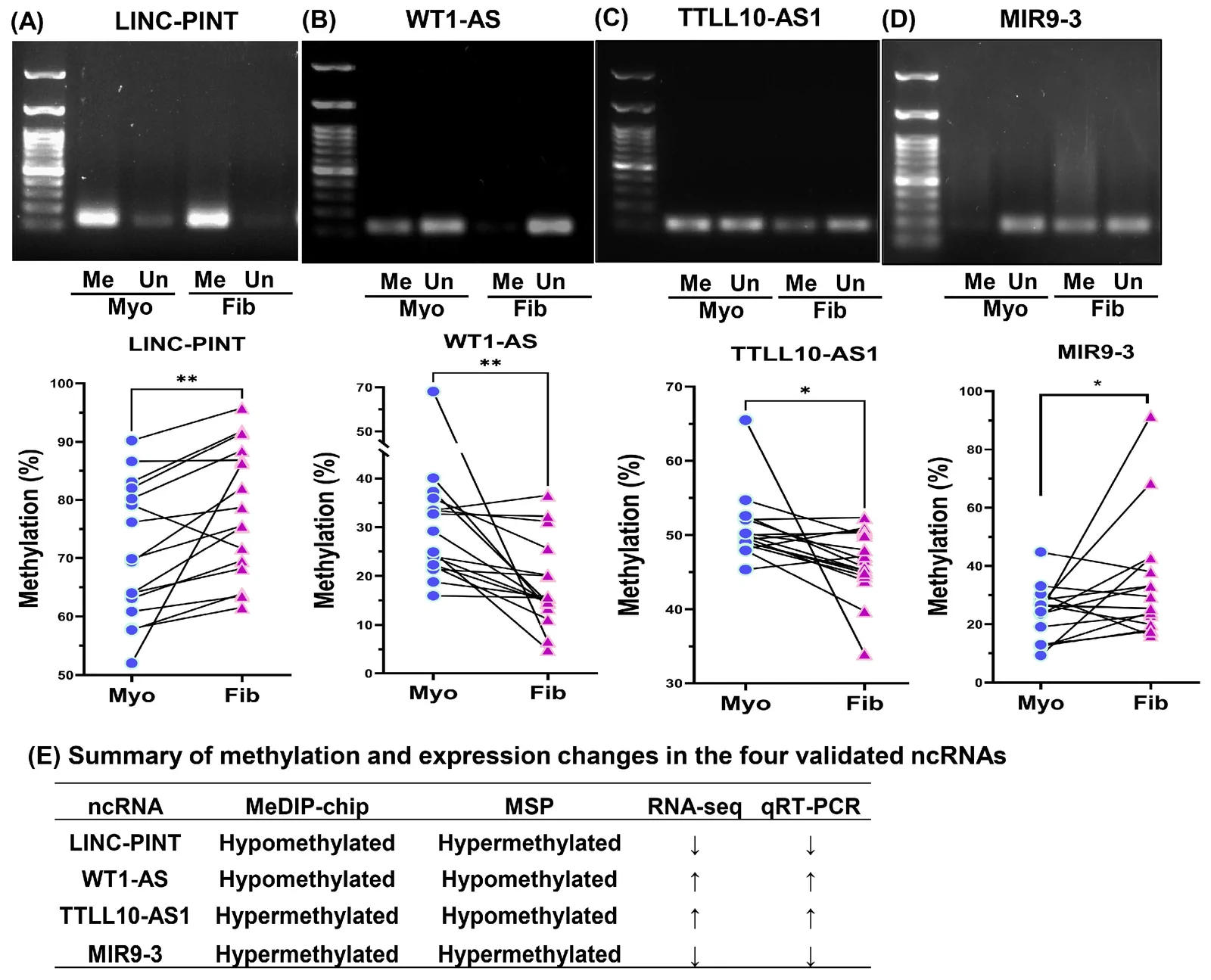
Validation of promoter methylation of selected ncRNAs by methylation-specific PCR (MSP). Representative MSP analysis of the promoter regions of LINC-PINT (**A**), WT1-AS (**B**), TTLL10-AS1 (**C**), and MIR9-3 (**D**) in paired fibroid and myometrium tissues (*n* = 16 pairs). Representative gel images are shown above the quantitative analysis. Bands corresponding to methylated (Me) and unmethylated (Un) alleles were quantified using ImageJ software version 1.54k. The percentage of methylation was calculated as Me/(Me + Un) × 100. Each line connects matched myometrial (Myo) and fibroid (Fib) samples from the same patient. Data are presented as mean ± SEM. Statistical significance is indicated by asterisks (* *p* < 0.05; ** *p* < 0.01). (**E**) Summary comparison of promoter methylation changes determined by MeDIP–chip and MSP and expression changes determined by RNA-seq and qRT-PCR for the four selected ncRNAs. Upward and downward arrows indicate increased and decreased expression, respectively, in fibroids relative to matched myometrium.

To validate selected candidates based on multiple criteria including significant promoter methylation identified by MeDIP–chip, CpG-rich promoter architecture, sufficient probe coverage, feasibility of MSP primer design, detectable transcript expression by qRT-PCR, and potential biological relevance based on the published literature, four ncRNAs with high GC content and CpG density in their promoter regions were selected from the MeDIP–chip dataset, two hypomethylated lncRNAs (LINC-PINT and WT1-AS) and two hypermethylated ncRNAs (TTLL10-AS1 and MIR9-3), and analyzed by qRT-PCR. Fibroids exhibited increased expression of WT1-AS and TTLL10-AS1 and decreased expression of LINC-PINT and MIR9-3 relative to matched myometrium (Figure 6A). Stratification by MED12 mutation status showed that LINC-PINT was significantly downregulated, whereas WT1-AS was significantly upregulated, in MED12-mutant fibroids compared with wild-type tumors (Figure 6B). The expression levels of LINC-PINT and TTLL10-AS1 as determined by qRT-PCR did not correspond to their promoter methylation patterns identified by MeDIP–chip. To further validate the methylation status of select genes, bisulfite conversion followed by methylation-specific PCR was performed in paired samples (*n* = 16 pairs) (Figure 7). Statistical analysis confirmed significant hypermethylation of LINC-PINT and MIR9-3 and hypomethylation of WT1-AS and TTLL10-AS1 in fibroids compared with matched myometrium (Figure 7A–D). A comparison of the MeDIP–chip, MSP, RNA-seq, and qRT-PCR findings for the four selected ncRNAs is summarized in Figure 7E.

## 3. Discussion

The present study provides a comprehensive genome-wide characterization of promoter methylation patterns of ncRNAs in uterine fibroids using MeDIP–chip analysis combined with targeted methylation and expression validation. We identified widespread alterations in promoter methylation affecting both lncRNAs and miRNAs in fibroids compared with matched myometrium, supporting the concept that epigenetic dysregulation extends beyond protein-coding genes to include multiple classes of regulatory ncRNAs. Validation of four selected ncRNA candidates by methylation-specific PCR indicated hypomethylation of WT1-AS and TTLL10-AS1 and hypermethylation of LINC-PINT and MIR9-3 in fibroids. However, the methylation patterns observed for LINC-PINT and TTLL10-AS1 did not correspond with those identified by the MeDIP–chip analysis. These findings suggest that although promoter methylation may contribute to the regulation of selected ncRNAs in fibroids, it does not uniformly predict transcriptional outcomes. Instead, the expression of ncRNAs in fibroids is likely influenced by multiple regulatory layers, including locus-specific epigenetic modifications and other transcriptional or post-transcriptional mechanisms.

Epigenetic regulation has emerged as an important contributor to the molecular pathogenesis of uterine fibroids [40]. In addition to alterations in protein-coding genes, accumulating evidence indicates that epigenetic mechanisms, particularly DNA methylation, also influence the expression of non-coding RNAs, including lncRNAs and miRNAs [41]. Consistent with these observations, the present study identified numerous ncRNA promoters with altered methylation patterns in fibroids compared with matched myometrium. However, the relationship between promoter methylation and transcript expression was not uniformly consistent across all candidates. This finding suggests that although DNA methylation represents an important regulatory mechanism, it does not fully account for ncRNA dysregulation in fibroids. Instead, ncRNA expression is likely shaped by a combination of epigenetic and transcriptional mechanisms, including chromatin remodeling, transcription factor binding, enhancer activity, histone modifications, and post-transcriptional regulation [24], which together may influence the gene expression networks involved in fibroid growth and extracellular matrix remodeling. The discrepancies observed between the MeDIP–chip results and methylation-specific PCR (MSP) validation for some ncRNAs are likely attributable to methodological differences between these approaches. MeDIP–chip detects regions enriched for methylated DNA using antibodies against 5-methylcytosine and therefore reflects regional methylation patterns across relatively broad genomic intervals [42], whereas MSP provides locus-specific assessment of individual CpG sites following bisulfite conversion [43]. Because DNA methylation within CpG-rich promoter regions is often heterogeneous, methylation signals detected by MeDIP–chip may not precisely correspond to the CpG sites targeted by MSP primers. In addition, the sensitivity of MeDIP-based approaches can be influenced by factors such as CpG density and antibody enrichment efficiency, which may contribute to differences between array-based and locus-specific measurements. These technical considerations likely explain the discordance observed for LINC-PINT and TTLL10-AS1 between the genome-wide methylation profiling and targeted validation analyses. Consistent with this complexity, directional analysis showed that 40 of 69 differentially expressed loci (58.0%) exhibited an inverse relationship between promoter methylation and expression, whereas 29 (42.0%) showed a non-inverse relationship. Thus, although a subset of ncRNAs exhibited the canonical inverse methylation–expression pattern, this relationship was not universal. These findings support the possibility of locus-specific methylation-associated regulation while also indicating substantial contributions from methylation-independent regulatory mechanisms. Importantly, these findings highlight the necessity of locus-specific validation when interpreting genome-wide methylation datasets and emphasize the complexity of epigenetic regulation of ncRNAs in fibroids. Despite these methodological differences, the ncRNAs validated in this study remain biologically relevant candidates that may participate in epigenetically mediated regulatory networks contributing to fibroid development.

Among the ncRNAs identified in this study, LINC-PINT, WT1-AS, TTLL10-AS1, and MIR9-3 have been implicated in tumor biology and may also be relevant to fibroid pathogenesis. LINC-PINT has been widely described as a tumor-suppressive lncRNA involved in the regulation of cell proliferation and apoptosis, and is frequently downregulated in several malignancies [44]. Mechanistically, in melanoma, LINC-PINT can interact with epigenetic regulators such as EZH2, recruiting this histone methyltransferase to target gene promoters to mediate transcriptional repression [45]. Notably, EZH2 is reported to be overexpressed in uterine fibroids and has been shown to promote fibroid cell proliferation and extracellular matrix production through activation of pathways such as Wnt/β-catenin signaling [46,47,48]. Thus, reduced expression of LINC-PINT, potentially mediated by promoter hypermethylation, could contribute to fibroid growth by altering EZH2-associated regulatory networks. Similarly, the WT1 locus and its antisense transcript WT1-AS have been shown to participate in tumor-related signaling pathways and may influence cell cycle regulation and apoptosis through interactions with targets such as WT1, PIK3AP1, and p53 [49,50,51]. Dysregulation of WT1-AS expression has been reported in several malignancies, suggesting that this transcript may function as a regulator of proliferation in a context-dependent manner [52,53,54,55]. Although the functional role of TTLL10-AS1 remains less well characterized, emerging studies suggest that it may participate in post-transcriptional regulatory networks involved in cancer progression [56]. MIR9-3 belongs to the miR-9 family, is often silenced by methylation in certain cancers, such as chronic lymphocytic leukemia and breast cancer, acts as a tumor suppressor, and participates in diverse biological processes including cell proliferation and migration [57,58]. Overexpression of miR-9-3 led to suppressed cell proliferation and enhanced apoptosis, together with downregulation of NFκB1 in I83-E95 cells [57]. Taken together, the altered expression of these ncRNAs in fibroids suggests that they may participate in key pathogenic processes such as abnormal cell proliferation, extracellular matrix accumulation, and activation of signaling pathways including Wnt/β-catenin and NF-κB, which are central features of fibroid development [39,59]. Notably, the pathways enriched among ncRNAs with altered promoter methylation in the present study are consistent with biological processes reported in prior genome-wide methylation analyses of fibroids that focused on protein-coding genes. For example, integrative methylome analyses have shown that differentially methylated genes in fibroids are significantly enriched in cancer-related processes and reproductive system disease pathways [13]. Similarly, another genome-wide methylation study reported that both hypomethylated/upregulated and hypermethylated/downregulated genes were predominantly associated with cancer-related pathways and extracellular matrix-related genes, including collagen family members [60]. The overlap between these coding gene-based analyses and our ncRNA-centered findings, which highlight enrichment of cancer-associated signaling, PI3K/AKT pathways, hormone-responsive networks, and extracellular matrix-associated processes, supports the concept that epigenetic dysregulation across both coding and non-coding transcripts contributes to the regulatory networks driving fibroid pathogenesis.

Fibroid development is driven by a complex interplay of hormonal, genetic, and epigenetic factors [61]. Previous studies have identified key contributors to fibroid pathogenesis, including MED12 mutations, dysregulated signaling pathways, and excessive extracellular matrix accumulation, yet the regulatory mechanisms connecting these processes remain incompletely understood [39,62]. Non-coding RNAs represent an additional layer of gene regulation capable of coordinating multiple biological pathways through transcriptional, post-transcriptional, and epigenetic mechanisms [63,64]. In the present study, the altered promoter methylation patterns identified in fibroids, together with the dysregulated expression of selected ncRNAs, suggest that epigenetically regulated ncRNA networks may contribute to the molecular landscape underlying fibroid development. Because ncRNAs often function as central regulators of gene expression programs, epigenetic modifications affecting their promoter regions could have broad downstream effects on pathways controlling cell proliferation, extracellular matrix remodeling, and tissue fibrosis, hallmark features of fibroid growth. Importantly, epigenetic modifications such as DNA methylation are potentially reversible, raising the possibility that targeting ncRNA-associated epigenetic pathways may provide new opportunities for therapeutic intervention. Whether these methylation signatures can distinguish uterine fibroids from other uterine tumors remains unknown and will require validation in independent cohorts that include other benign and malignant uterine lesions. Future studies will therefore be necessary to define the functional roles of these ncRNAs in fibroid biology and to evaluate their potential utility as biomarkers or therapeutic targets.

Several limitations of this study should be acknowledged. First, the discovery MeDIP–chip cohort was relatively small, although the principal observations were subsequently evaluated by MSP and expression analysis in substantially larger independent validation cohorts. Second, MeDIP–chip provides regional methylation information with lower genomic resolution than bisulfite sequencing-based approaches. Future studies employing whole-genome bisulfite sequencing or targeted bisulfite sequencing may provide more comprehensive characterization of locus-specific methylation patterns. Third, MED12 mutation analysis was performed only in the larger validation cohort because adequate statistical power was available. In contrast, the MeDIP–chip discovery cohort (*n* = 8 pairs) and MSP validation cohort (*n* = 16 pairs) became too small after subdivision into MED12-mutant and wild-type groups to permit meaningful subgroup analysis; future studies using larger methylation datasets will be necessary to determine whether promoter methylation differs according to MED12 status. In addition, the MeDIP–chip analysis used the manufacturer’s standard peak-calling pipeline based on the NimbleScan sliding-window algorithm and a predefined significance threshold rather than a genome-wide false discovery rate correction. Although this approach may increase the likelihood of false-positive discoveries, the principal candidate loci identified in the discovery analysis were subsequently evaluated by independent MSP and expression validation in larger patient cohorts. Finally, the present study was designed as a genome-wide profiling investigation and therefore does not establish direct causal relationships between promoter methylation and ncRNA function. Functional studies examining whether manipulation of promoter methylation alters ncRNA expression and fibroid biology will be important future investigations.

In summary, our findings demonstrate that genome-wide promoter methylation profiles do not consistently predict ncRNA expression in uterine fibroids, highlighting the complexity of epigenetic regulation beyond DNA methylation alone. Although promoter methylation may contribute to the regulation of selected ncRNAs, our results indicate that additional epigenetic and transcriptional mechanisms, including chromatin remodeling, histone modifications, transcription factor activity, and post-transcriptional regulation, are likely involved in shaping the ncRNA landscape in fibroids. These findings underscore the importance of locus-specific validation when interpreting genome-wide methylation datasets and provide a foundation for future studies investigating integrated epigenetic regulatory networks and evaluating ncRNAs as potential biomarkers or therapeutic targets for uterine fibroids.

## 4. Materials and Methods

### 4.1. Myometrium and Fibroid Tissues Collection

Surgically resected intramural uterine fibroids (2–5 cm in diameter) and paired myometrial tissues were obtained from women undergoing gynecologic surgery at Harbor UCLA Medical Center (*n* = 68–94 pairs). All study procedures were approved by the Institutional Review Board of The Lundquist Institute at Harbor UCLA Medical Center (IRB #18CR-31752-01R), and written informed consent was obtained from all participants prior to surgery. None of the patients had received hormonal therapy for at least three months before tissue collection. Donor ages ranged from 30 to 49 years (mean ± SD: 43 ± 3.9 years). Immediately following surgical excision, tissue specimens were snap-frozen in liquid nitrogen and stored under cryogenic conditions until further processing, according to established protocols [7,8].

### 4.2. MeDIP–Chip Microarray and Bioinformatic Analysis

Genome-wide DNA methylation profiling was performed by Arraystar Inc. (Rockville, MD, USA). Briefly, genomic DNA was isolated using the DNeasy Blood & Tissue Kit (Qiagen, Fremont, CA, USA), quantified, and quality-assessed using a NanoDrop ND-1000 spectrophotometer. Purified genomic DNA was fragmented by sonication to an average size of 200–1000 bp. One microgram of fragmented DNA was subjected to methylated DNA immunoprecipitation (MeDIP) using a mouse monoclonal anti-5-methylcytosine antibody (Diagenode, Denville, NJ, USA). Immunoprecipitated DNA was purified using Qiagen MinElute columns, while corresponding input DNA served as a reference control. Input and MeDIP-enriched DNA were labeled with Cy3- and Cy5-conjugated random 9-mers, respectively, and co-hybridized to Arraystar Human ncRNA Promoter Microarrays. Each array interrogated 27,248 lncRNA promoters (−1300 bp to +500 bp relative to transcription start sites) and 622 miRNA promoters (up to −50 kb upstream of mature miRNA loci), represented by approximately 180,000 probes. Arrays were scanned using an Agilent G2505C scanner (ALT, East Lyme, CT, USA) and raw signal intensities were extracted using Agilent Feature Extraction software (v11.0.1.1). Data were median-centered, quantile-normalized, and linearly smoothed using the Ringo (v1.58.0), limma (v3.50.0), and MEDME (v1.54.0) Bioconductor packages. Enriched methylation peaks were identified from normalized log_2_ ratio data using a sliding-window peak detection algorithm (NimbleScan v2.5) with the following parameters: window width of 1500 bp, minimum of two probes per peak, −log_10_(*p*) ≥ 2, and maximum probe spacing of 500 bp. Functional enrichment analyses were performed using RNAenrich [65]. Microarray data were deposited in the Gene Expression Omnibus (GEO) under accession number GSE324138.

### 4.3. RNA Isolation and qPCR Analysis

Total RNA was isolated from fibroid and matched myometrial tissues using TRIzol reagent (Thermo Fisher Scientific, Waltham, MA, USA) according to the manufacturer’s instructions. RNA concentration and integrity were assessed using a NanoDrop 2000c spectrophotometer (Thermo Scientific, Wilmington, DE, USA) and an Agilent 2100 Bioanalyzer (Agilent Technologies, Santa Clara, CA, USA), as previously described [39]. For lncRNA analysis, 2 μg of total RNA was reverse-transcribed using random hexamer primers following the manufacturer’s protocol (Applied Biosystems, Carlsbad, CA, USA). For miRNA detection, primer design and PCR conditions were performed as previously reported [66]. Quantitative PCR was conducted using SYBR Gene Expression Master Mix (Applied Biosystems) on an Applied Biosystems StepOne Real-Time PCR System. Thermal cycling conditions consisted of an initial incubation at 95 °C for 10 min, followed by 40 cycles of denaturation at 95 °C for 15 s and annealing/extension at 60 °C for 1 min. All reactions were performed in triplicate. Relative expression levels of lncRNAs were normalized to FBXW2, while RNU6-2 served as the internal control for miRNA analysis. Gene expression was calculated using the comparative cycle threshold (2^−ΔΔCq^) method, as recommended by the manufacturer. Primer sequences used in this study were as follows: LINC-PINT (Forward, 5′-GCCCAGAGCTGTGGTATAAAT-3′; Reverse, 5′-CATGGTTGAGAGCAGGTGTAT-3′); WT1-AS (Forward, 5′-TGGTCTGAACCACCGATTG-3′; Reverse, 5′-TCCATAGACTGCCTCTCCTT-3′); TTLL10-AS1 (Forward, 5′-ATTCGTGCCGGGATGATATG-3′; Reverse, 5′-TGTGCTTCCGCTTTCTAACT-3′); FBXW2 (Forward, 5′-CCTCGTCTCTAAACAGTGGAATAA-3′; Reverse, 5′-GCGTCCTGAACAGAATCATCTA-3′); MIR9-3 (Forward, 5′-GCAGTCTTTGGTTATCTAGCTG-3′; Reverse, 5′-GGTCCAGTTTTTTTTTTTTTTTCATAC-3′); RNU6-2 (Forward, 5′-CGCTTCGGCAGCACATATAC-3′; Reverse, 5′-AGGGGCCATGCTAATCTTCT-3′).

### 4.4. MED12 Mutation Analysis

Genomic DNA was isolated from fibroid tissues and paired myometrium (100 mg per sample) using the MagaZorb DNA Mini-Prep Kit (Promega, Madison, WI, USA) according to the manufacturer’s instructions. MED12 exon 2 was amplified by PCR using the following primers (5′–3′): forward, GCCCTTTCACCTTGTTCCTT, and reverse, TGTCCCTATAAGTCTTCCCAACC. PCR products were subjected to Sanger sequencing (Laragen Inc., Culver City, CA, USA) using BigDye Terminator v3.1 chemistry. Sequence chromatograms were analyzed using ChromasPro software (version 2.1.8) and aligned to the MED12 reference genomic (NG_012808) and transcript (NM_005120) sequences to identify mutations.

### 4.5. Bisulfite Conversion and Methylation-Specific PCR

Genomic DNA was extracted from fibroid and matched myometrium samples (*n* = 16 pairs) and subjected to bisulfite conversion using the EZ DNA Methylation Kit (Zymo Research Corporation, Irvine, CA, USA), according to the manufacturer’s instructions. Bisulfite treatment converts unmethylated cytosines to uracil while leaving methylated cytosines unchanged, thereby enabling discrimination between methylated and unmethylated DNA sequences using methylation-specific PCR (MSP) [67]. The resulting bisulfite-converted DNA was immediately used for methylation-specific PCR. Methylation-specific primers were designed to amplify methylated (Me) and unmethylated (Un) promoter regions of LINC-PINT, WT1-AS, TTLL10-AS1, and MIR9-3. Primer sequences were as follows: LINC-PINT (Me: Forward, 5′-GTTTTTTTTATTTTTTTCGAGTTTC-3′; Reverse, 5′-TCGATATTTACCTCTAACCCTTCG-3′; Un: Forward, 5′-TTTTTTTTATTTTTTTTGAGTTTTG-3′; Reverse, 5′-CAATATTTACCTCTAACCCTTCACT-3′); WT1-AS (Me: Forward, 5′-TTTAATTTTTTTACGGTTAAGTCGG-3′; Reverse, 5′-AACCACAAACAATACTCGACGA-3′; Un: Forward, 5′-TTTAATTTTTTTATGGTTAAGTTGG-3′; Reverse, 5′-AAAACCACAAACAATACTCAACAAA-3′); TTLL10-AS1 (Me: Forward, 5′-GAGTGAGAATAGGGATTCGATTAC-3′; Reverse, 5′-TAACCCGAACCAAACTCTACGTA-3′; Un: Forward, 5′-GGAGTGAGAATAGGGATTTGATTAT-3′; Reverse, 5′-CCTAACCCAAACCAAACTCTACATA-3′); MIR9-3 (Me: Forward, 5′-GGTTTTTGTTTTCGGTTTTTTC-3′; Reverse, 5′-ACCTCAAAACTACGCACCGT-3′; Un: Forward, 5′-GGTTTTTGTTTTTGGTTTTTTTGT-3′; Reverse, 5′-AACCTCAAAACTACACACCATC-3′). PCR amplification was performed using HotStarTaq Plus PCR Master Mix (Qiagen, Valencia, CA, USA) under the following conditions: initial denaturation at 95 °C for 5 min, followed by 38 cycles of denaturation at 94 °C for 30 s, annealing at 58 °C for 30 s, and extension at 72 °C for 1 min. PCR products were resolved on 2% agarose gels and visualized under UV illumination.

### 4.6. Statistical Analysis

All data are presented as mean ± SEM and were analyzed using GraphPad Prism 11 software (GraphPad Software, San Diego, CA, USA). Data normality was assessed using the Kolmogorov–Smirnov, Shapiro–Wilk, D’Agostino–Pearson, and Anderson–Darling tests. As the datasets did not meet the assumptions of normality, non-parametric statistical tests were applied for all analyses. Comparisons between paired groups were assessed with the Wilcoxon matched-pairs signed-rank test, or the Mann–Whitney test for the unpaired two groups. Statistical significance was defined as a two-tailed *p* < 0.05.

## Data Availability

Raw data were generated at The Lundquist Institute. Derived data supporting the findings of this study are available from the corresponding author, O.K., on request.

## Supplementary Materials

The following supporting information can be downloaded at https://www.mdpi.com/article/10.3390/ijms27177781/s1.
